# Systemic immune responses after ischemic stroke: From the center to the periphery

**DOI:** 10.3389/fimmu.2022.911661

**Published:** 2022-09-20

**Authors:** Fan Wu, Zongchi Liu, Lihui Zhou, Di Ye, Yu Zhu, Kaiyuan Huang, Yuxiang Weng, Xiaoxing Xiong, Renya Zhan, Jian Shen

**Affiliations:** ^1^ Department of Neurosurgery, First Affiliated Hospital, College of Medicine, Zhejiang University, Hangzhou, China; ^2^ Department of Clinical Laboratory, Renmin Hospital, Faculty of Medical Sciences, Wuhan University, Wuhan, China

**Keywords:** ischemic stroke, immunosuppression, systemic immune response, neuro-immune crosstalk, immune system disorder, neuroendocrine system, autonomic nervous system, opportunistic infection

## Abstract

Ischemic stroke is a leading cause of disability and death. It imposes a heavy economic burden on individuals, families and society. The mortality rate of ischemic stroke has decreased with the help of thrombolytic drug therapy and intravascular intervention. However, the nerve damage caused by ischemia-reperfusion is long-lasting and followed by multiple organ dysfunction. In this process, the immune responses manifested by systemic inflammatory responses play an important role. It begins with neuroinflammation following ischemic stroke. The large number of inflammatory cells released after activation of immune cells in the lesion area, along with the deactivated neuroendocrine and autonomic nervous systems, link the center with the periphery. With the activation of systemic immunity and the emergence of immunosuppression, peripheral organs become the second “battlefield” of the immune response after ischemic stroke and gradually become dysfunctional and lead to an adverse prognosis. The purpose of this review was to describe the systemic immune responses after ischemic stroke. We hope to provide new ideas for future research and clinical treatments to improve patient outcomes and quality of life.

## 1 Introduction: From the center to the periphery

Stroke is a common disease worldwide, characterized by a high incidence, disability rate and mortality, that poses a notable threat to human health (1). Stroke is divided into ischemic stroke and hemorrhagic stroke, which present distinct pathological changes. Thus, different treatment plans should be selected according to different conditions. Ischemic stroke is caused by an embolus or local thrombosis when it interrupts the blood flow to some areas of the brain (2, 3). On the contrary, cerebral ischemia in hemorrhagic stroke is caused by rupture of the responsible artery or vein. About 80% of strokes are reported to be ischemic (4). Unlike hemorrhagic stroke, ischemic stroke is often insidious and arises without warning. When obvious neurological symptoms appear, it means that nerve damage has occurred, which can become irreversible (5, 6). In addition to neurological damage, patients with ischemic stroke also suffer from multiple systemic complications (7). However, the mechanisms of multiple organ dysfunction after ischemic stroke are complex. Nevertheless, long-term studies have indicated that the systemic inflammation caused by immune system disorder is involved in the pathophysiological mechanism of these complications (8).

For a long time, the nervous and immune systems have been studied independently. Because of the blood-brain barrier (BBB), the entire central nervous system (CNS) seems to be separated from the peripheral immune system. However, no system can exist and operate independently. The nervous and immune systems interact with and regulate each other from the embryonic stage (9, 10). As research has progressed, the classical concept of separation of the CNS and peripheral immune system is gradually giving way to dynamic intermodulation (11).

Normally, under the protection offered by the BBB constructed by endothelial cells, the end-feet of astrocytes, and pericytes embedded in the basement membrane of capillaries, microglia play a key role in immunosurveillance as resident brain macrophages (12–14). When ischemic stroke occurs, the brain tissue responsible for the affected blood vessels is hypoxic and damaged, and even dies. The damage-associated molecular patterns (DAMPs) released by dying cells trigger a cascade of signals that activates the innate immune system (15, 16). These DAMPs stimulate microglial activation and polarize into phagocytic, proinflammatory phenotypes, releasing a large number of proinflammatory factors (17). The presence of a large number of inflammatory factors, known as an inflammatory cytokine storm, damage nerve cells and the BBB and attracts peripheral immune cells to infiltrate the lesion area (18). Many inflammatory cytokines circulate through the bloodstream into the periphery, resulting in a cytokine storm that can cause peripheral organ dysfunction and further aggravate systemic inflammation. Meanwhile, the neuroendocrine system (such as the hypothalamic-pituitary-adrenal (HPA) axis, as well as the autonomic nervous system, are deactivated after onset (9). On the one hand, their dysfunction manifests itself in the abnormal function of target organs. On the other hand, due to the close relationship with the immune system, their abnormal function can often cause immune system disorders, such as immunosuppression. As such, they may contribute to the process of neuroimmune crosstalk after ischemic stroke and link the center to the periphery (19). In conclusion, the central-peripheral crosstalk after the onset of ischemic stroke begins with neuroinflammation and is mediated by neural and humoral regulatory pathways, linking the central and peripheral regions. The existence of this crosstalk disturbs the normal operation of many organs and leads to poor prognosis.

In this review, we explore the central-peripheral crosstalk after ischemic stroke with neuroinflammation as the entry point and neural and humoral regulatory pathways as the clues. Subsequently, we explore the consequences of this crosstalk. In these discussions, we also touch on systemic immune system activation and immunosuppression after crosstalk. We hope our work will provide insights for researchers in the field and contribute to the development of new treatments in the future.

## 2 The bridgehead: Neuroinflammation

**Figure 1:**
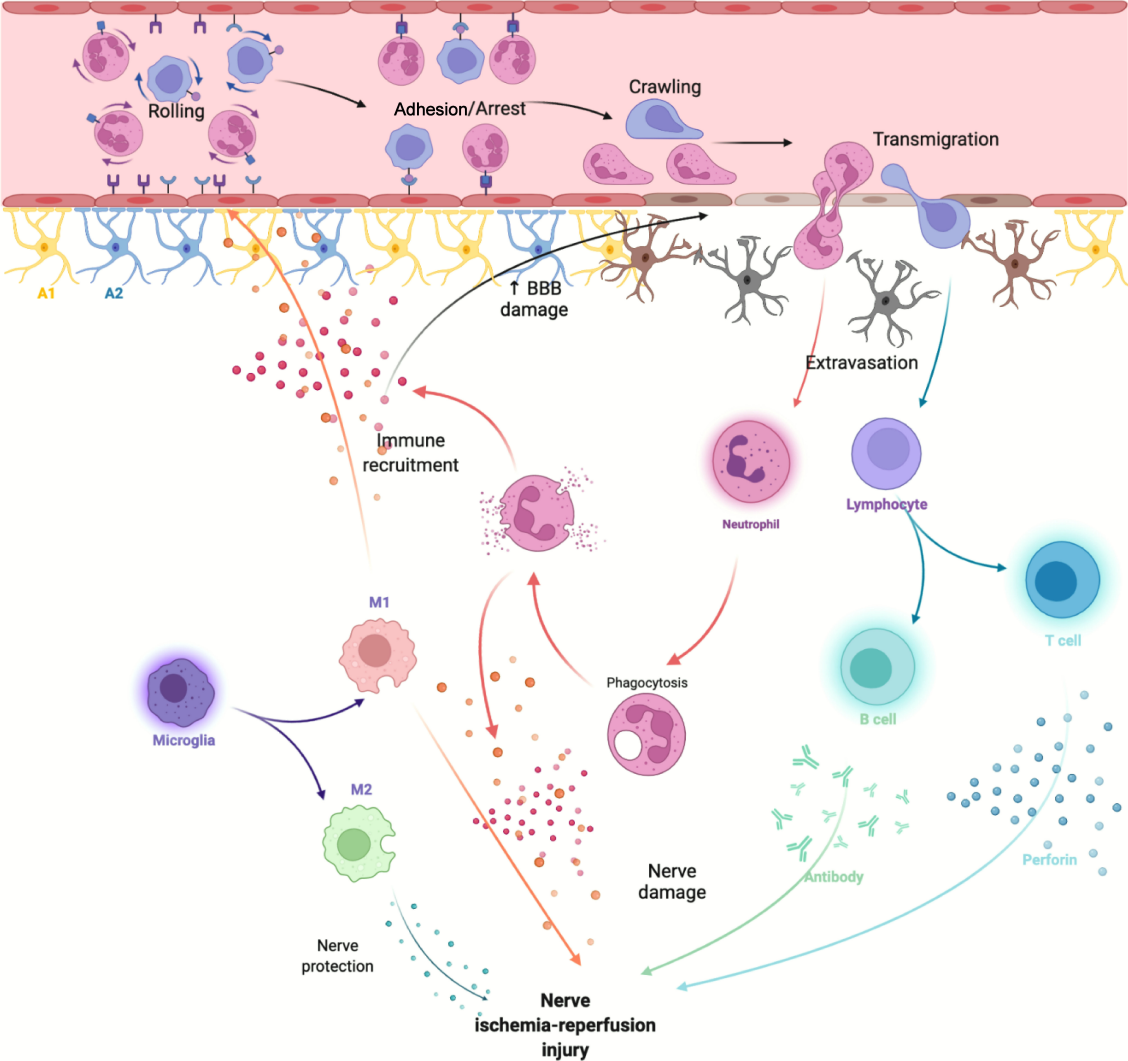
After cerebral ischemia-reperfusion injury, DAMPs released by necrotic cells activate innate immune cells in the CNS and attract peripheral immune cells to activate adaptive immune responses.

Neuroinflammatory response after ischemic stroke is involved in both early nerve damage and late nerve repair. Microglia, monocytes and neutrophils in the innate immune system, along with astrocytes, traditionally considered to be the major destructive factors after stroke, are also extensively involved in brain repair after stroke. T and B cells in the adaptive immune system have roles in CNS damage and repair (20) (**Figure 1**).

### 2.1 Microglia

Microglia, as resident CNS macrophages, are the first line of defense after brain damage (21). Under physiological conditions, they play an important role in maintaining CNS homeostasis and have no or only physiologically required phagocytic function (22). Once brain damages occur, as nerve cells in the central area of infarction die, a large number of DAMPs are released and activate microglia (23).

Under physiological conditions, microglia show a resting state characterized by a ramified morphology. With activation, morphological changes in microglia are observed, accompanied by upregulated expression of various cell surface markers (24). In animal models, we can see that their numbers and phenotypes change dynamically as the disease progresses. In a model of permanent middle cerebral artery occlusion (MCAO), activated microglia were detected at the boundary of ischemic lesions 30 min after stroke (25). In a photothrombotic stroke model, neurons in the core lesion died within 2 h, accompanied by activation of astrocytes and microglia (26). Despite a significant decrease in the number of microglia in the core after stroke, the number of microglia in the ischemic core and the boundary zone increased within hours after IS and peaked at 2–3 days (27, 28). At this stage, microglial responses can be characterized by changes in morphology and act like amoeba (29). After transient MCAO, these amoeboid microglia begin to appear in ischemic lesions 2–10 h after reperfusion. These amoeba-like microglia, along with rounded microglia, predominate in core lesions and mingle with highly branched microglia to the boundary 22 h after reperfusion (30, 31). Subsequently, amoeboid microglia in the core are further enriched 3–7 days after stroke (30). In the photothrombotic model, more amoeba cells infiltrate into the core by day 4. By day 7, amoeba cells are present throughout the lesion. However, over time, when the course of the disease enters the chronic stage, only a small number of deformed cells are found in the peripheral and distal areas of the infarct, and the microglial response decreases (27).

In addition to morphological changes, the phenotype of microglia also changes during this pathophysiological process. It is noteworthy that the activated microglia in animal models with different functions can be divided into M1 phenotype (proinflammatory) and M2 phenotype (anti-inflammatory), which are involved in tissue damage and repair, respectively (22, 32). The M2 phenotype can be further subdivided into three subtypes (M2a, M2b and M2c) (33). Microglial activation and different phenotypes can be determined by detecting different surface markers. The classical markers of M1 microglia include integrin alpha-M (CD11b), CD16, CD32 and CD86, while the classical markers of M2 microglia include macrophage mannose receptor 1 (CD206) (34).

After ischemic stroke, Toll-like receptor (TLR)4 on the surface of microglia recognizes and binds with high mobility group box (HMGB)1 protein, heat shock protein, purine and other substances (21). This promotes activation of the nuclear factor (NF)-κB pathway and the M1 phenotype transformation of microglia, which exists in a proinflammatory state and secretes proinflammatory cytokines (35). Overexpression of signal transducer and activator of transcription (STAT)1 and interferon (IFN) regulatory factor 5 is correlated with M1 polarization (36, 37). In mice, the number of TLR4^+^ cells, as well as NF-κB^+^ and interleukin (IL)-1β^+^ cells, increases significantly 72 h after MCAO (38). At this stage, M1 microglia primarily serve a detrimental role by damaging the BBB, aggravating brain edema and promoting neuronal apoptosis by producing and secreting a large number of inflammatory mediators (18). In mouse models, tumor necrosis factor (TNF)-α secreted by M1 microglia increases endothelial necrosis and BBB leakage and further promotes neuroinflammation and cerebral edema, leading to poor outcomes, while increased IL-17A levels exacerbate neuronal death (39, 40). Expression of chemokines and ligands increases, attracting peripheral immune cells to the lesion area and exacerbating the inflammatory response (41).

In contrast, the M2 phenotype, primarily as a protective phenotype, promotes resolution of inflammation by secreting IL-4, IL-10 and transforming growth factor (TGF)-β, thereby indirectly preventing inflammation-induced BBB destruction. IL-4 and IL-10 inhibit the expression of IFN-γ, TNF-α and IL-1β by inhibiting activation of the NF-κB signaling pathway, while increasing expression of other anti-inflammatory factors (42). IL-10 plays a protective role by limiting the expression and activity of matrix metalloproteinase (MMP) by protecting endothelial cells (42, 43). TGF-β decreases the levels of TNF-α and monocyte chemoattractant protein-1 through the ALK5-p-Smad2/3 signaling pathway (44).

It is important to note that this dichotomy is not perfect because these two phenotypes have many overlapping functions. The M1 phenotype can also help the repair process, but the M2 phenotype can also cause damage. Whether microglia promote damage or repair depends on the stimuli they receive (45). That is why the spatiotemporal phenotypic variation has puzzled researchers for a long time. After analyzing changes in the expression of M1 and M2 markers, researchers have concluded that the M1/M2 expression pattern changes dynamically after IS. The M2 phenotype is beneficial in the early stage and then changes to harmful M1 phenotype in the late stage (34). This microglia-targeting therapy provides insight that promotes early M2 phenotype transformation and inhibits late M1 phenotype transformation.

### 2.2 Astrocytes

As the most abundant glial cells in the brain, astrocytes play a CNS “steward” role in providing metabolic and nutritional support, regulating synaptogenesis, ion homeostasis, neurotransmitter buffering, maintaining the integrity of the BBB, and promoting neural network activity patterns (46).

Astrocytes play a dual role in the pathophysiology of ischemic stroke. Within minutes of onset, the astrocytes activate and multiply in response to various inflammatory factors released by ischemic/hypoxic cells. In this process, known as reactive astrocyte hyperplasia, astrocytes exhibit cell hypertrophy, proliferation, and increased expression of intermediate proteins including glial fibrillary acidic protein (GFAP), vimentin, and nestin (47). Among these markers, the most prominent is upregulation of intermediate proteins, especially GFAP, which is the main component of the intermediate system of adult astrocytes (48, 49). Serum GFAP level is positively correlated with the National Institutional Health Stroke Scale (NIHSS), and may predict worse prognosis (50). Within a few days, reactive astrocytes form glial scars around ischemic lesions in the brain and confine the inflammatory response locally (51, 52). The process of reactive astrocyte proliferation is dynamic. Mild glial scars fade over time. However, if the lesion is more extensive, more significant glial scars may be permanent (53). With the accumulation of glial scars, they go from helpers limiting inflammatory progression to obstacles to axon growth and neuronal repair (54–56).

As research progresses, astrocytes are not only bystanders of the immune response after ischemic stroke, but also deeply involved in the inflammatory response after ischemic stroke. After reactive proliferation, astrocytes produce and release various proinflammatory mediators, such as IL-6, TNF-α, IL-1α, IL-1β and IFN-γ, as well as free radicals (57). Astrocytes, on the one hand, damage the integrity of BBB and aggravate neuron damage through these proinflammatory factors. On the other hand, they interact with microglia and recruit peripheral immune cells to participate in the inflammatory response (57).

In the study of astrocyte and microglia interactions, astrocytes induced by IL-1α, TNF-α and complement component C1q secreted by activated microglia are classified as A1 subtype. Although A1 subtype astrocytes have lost their ability to promote neuronal survival, growth, synaptic formation, and phagocytosis, they induce neuronal and oligodendrocyte death (58). In contrast, the A2 subtype is considered protective. This subtype secretes IL-2, IL-10 and TGF-β, which accelerates inflammation resolution. In particular, reactive astrocytes after transient MCAO show increased transcription associated with the A2 subtype by transcriptome analysis. This suggests that the A2 subtype is more involved in inflammatory suppression and glial scar formation (59).

A recent study found that the reactive-oxygen-species-mediated NF-κB/STAT3 pathway can inhibit activation of A1 subtype astrocytes and increase activation of A2 subtype, exerting neuroprotective effects (60). Although future treatment regimens that reduce the A1 subtype and increase the A2 subtype will help improve the outcome of IS patients, it should be noted that the excessive accumulation of the A2 subtype may lead to the accumulation of glial scars, which may lead to adverse results.

### 2.3 Neutrophils

Neutrophils are the first peripheral immune cells to respond to ischemic brain injury (61). They infiltrate the ischemic area a few hours after cerebral ischemia occurs, and accumulation of neutrophils may peak in the first 3 days (62). Ischemic brain tissue releases a large number of DAMPs that activate microglia and astrocytes to release proinflammatory mediators (e.g. IL-1β and TNF) (63). Typically, chemokines including CXC chemokine ligand (CXCL)1, CXCL2 and CXCL5 (CXCL8 in humans) contribute to the release of neutrophils from bone marrow and recruitment to ischemic brain tissue (64). Expression of neutrophil chemokine receptors is increased to promote neutrophil activation (65). Animal studies have shown that using CXC receptor (CXCR)1 and CXCR2 inhibitors can significantly reduce neutrophil extravasation and infarct volume, and improve functional outcomes (66). However, inhibition of CXCR1 or CXCR2 alone does not reduce infarct size or improve function (67). Neutralizing the bioactivity of CXCL1 and CXCL2 only reduces neutrophil infiltration and does not reduce infarct size or improve neurological deficits (68).

Expression of many endothelial adhesion receptors on neutrophils is increased, including P-selectin glycoprotein ligand-1, lymphocyte function-associated antigen 1 and macrophage-1 antigen (69, 70). These components promote the adhesion and infiltration of neutrophils. This process is first mediated by selectin (P-selectin and E-selectin) to neutrophil rolling. Secondly, neutrophils migrate to the optimum anatomical location under the mediation of β2-integrins lymphocyte function-associated antigen 1 (αLβ2 integrin) and macrophage-1 antigen (αMβ2 integrin) (71). Activated neutrophils release various proteases (MMPs, elastase, cathepsin G and proteinase 3) and reactive oxygen species that strike the BBB with fatal force, allowing them to cross the endothelial layer (72). Finally, under the cascade of different chemical attractants, neutrophils reach the injured brain tissue and disrupt neural function by further disrupting the BBB through neutrophil extracellular traps and promoting thrombosis (64, 73, 74). Although treatment regimens targeting these molecules successfully interfere with neutrophil rolling and adhesion in animal models, they can effectively limit neutrophil accumulation and BBB leakage (18). However, clinical trials have been disappointing. Compared with placebo, there was no benefit and an increased risk of infection (74).

### 2.4 T cells

T cells originate in bone marrow, mature in the thymus and play a central role in the adaptive immune system (75). T cell transport marks the beginning of the T cell response in ischemic stroke (76). At present, there are three ways that T cells infiltrate pathological tissues: BBB, choroid plexus and meninges (77). After binding, rolling, stagnation and adhesion stages, T cells attach to endothelial cells by upregulating expression of specific adhesion molecules on the membrane. Initial adhesion and rolling of T cells are mediated by the binding between endoselectin and its ligand (78). Subsequently, integrins represented by very late antigen 4 and their ligands, such as vascular cell adhesion molecule-1, help the T cells to stay firmly on the endothelial cells (79). In animal models, during the first 24 h after MCAO, the CD3^+^ T cell margins were significantly infiltrated into the diseased tissue (80). However, the peak of T cell infiltration is correlated with disease severity. In the transient MCAO model, the peak occurs 3–5 days after induction. While, in permanent MCAO models, the peak is delayed, usually around 7 days after onset (76).

It is important to note that T cells perform different functions at different stages due to the existence of different functional subtypes. In the acute phase of ischemic stroke, T cells respond mainly in an antigen-independent manner and are closely related to the development of infarct volume (76). In animal models, TCR-transgenic mice bearing 1 single CD8^+^ (2C/RAG2, OTI/RAG1 mice) or CD4^+^ (OTII/RAG1, 2D2/RAG1 mice) T cell receptor (TCR), mice lacking accessory molecules of TCR stimulation (CD28^−/−^, PD1^−/−^, B7-H1^−/−^ mice) are as completely susceptible to ischemic reperfusion injury as wild-type mice are (81). In addition, certain T cell subtypes, including γδ T cells, do not naturally require antigenic stimulation for their activation (82). After 3–7 days, the T cell response gradually shifts to antigen-dependent with antigen recognition (83). The classic antigen-dependent T cell response involves two steps: initiation and reactivation. In ischemic stroke, soluble antigens leak into the blood and surrounding tissues due to the breakdown of the BBB. These soluble antigens promote the activation, proliferation and differentiation of naive T cells into effector cells after being treated by antigen-presenting cells (76). Through expression of TCR on the surface, T cells can be divided into CD8^+^ cytotoxic T cells and CD4^+^ helper T (Th) cells (84). Cytotoxic T cells directly destroy target cells by releasing cytotoxic perforin, granase and granulysin, or induce apoptosis by the Fas–Fas ligand pathway (85). Th cells bind to antigen-MHC class II molecules expressed on the surface of APCs, which are stimulated and can further differentiate into Th1, Th2, T regulatory (Treg) and other subtypes (86). Th1 cells secrete proinflammatory cytokines and Th2 cells produce anti-inflammatory cytokines (87). It is important to note that Treg cells, which are identified by expression of transcription factor forkhead box (FOX)P3, play a beneficial role in stroke (88, 89). Treg cells are rare in the early stages of the disease. On day 14 after stroke, FOXP3^+^ Treg cells account for 30–40% of CD4^+^ T cells, especially in and around the infarct area. In addition, the number of Treg cells continue to increase and last for up to 1 month thereafter (90). Treg cells work mainly in the late stages. They antagonize TNF-α and IFN-γ production from infiltrated immune cells, including microglia and T effector cells, by secreting IL-10 (91). Promotion and maintenance of the anti-inflammatory phenotype of microglia is another important mechanism by which Treg cells play a neuroprotective role (92). Hence, Treg cells could be an effective target for the treatment of stroke in the future (89).

At present, compared with other immune cells, the mechanism of T cells in ischemic stroke is still limited. Although it is thought to be deleterious in the early stages of ischemic stroke, its detailed function remains to be further defined (76).

### 2.5 B cells

At present, there are different and controversial views on the mechanism of B cells in ischemic stroke. Based on existing research, the distinct functions may be related to the time after stroke and different B cell subsets (93).

IL-10^+^ regulatory B cells, which account for only 0.5–0.7% of CD19^+^ B cells, have been shown to have CNS protective activity after stroke. Injection of all CD19^+^ B cells into the lesion site in B-cell-deficient mice reduced infarct volume 48 h later (94). Specifically, mice injected with IL-10^+^ B cells showed a decrease in infarct size and infiltrating cells, and a significant increase in Treg cells (95, 96). Some studies have shown that B cells do not play an important role in the acute phase of ischemic lesions, especially in the impact of infarct volume (97, 98).

B cells are either absent or harmful during functional recovery. The delayed deleterious effects of B cells are primarily seen in increased susceptibility to dementia, which appears a few weeks after stroke (99). In mouse models, activated B lymphocytes infiltrate infarcted tissue for several weeks after stroke. At the same time, IgM, IgG and IgA antibodies are found in nerve cells near the lesion. While directly impacting neuronal function, the accumulation of antibodies is associated with impairment in hippocampal long-term potentiation and leads to short-term memory deficit a few weeks after stroke (100).

Although the use of regulatory B cells cannot reduce brain injury in the acute stage, it can regulate long-term neurological dysfunction. However, the evidence from recent studies makes it difficult to clearly define the role of B cells in IS, and more studies are needed in the future.

## 3 Bridges

### 3.1 Inflammatory cytokines

All kinds of immune cells activated after the onset, whether proinflammatory or anti-inflammatory, will release a large number of inflammatory factors. These inflammatory factors are represented by ILs, TNFs and chemokines (101). They circulate to the periphery and become the “invisible hand” that connects the center to the periphery after an ischemic stroke.

#### 3.1.1 ILs

ILs can be broadly classified as anti-inflammatory and proinflammatory. IL-1, IL-8, IL-12, IL-15, IL-16, IL-20, IL-18 and IL-23/IL-17 play proinflammatory roles after ischemic stroke (102). IL-1, the best-known mediator of acute brain injury inflammation, is not present in patients with high levels of IL-1β in serum or plasma. IL-1 occurs in two forms: IL-1α (intracellular) and IL-1β (secreted) (16). After onset, increased IL-1β secretion activates phospholipase A2 to degrade arachidonic acid and destroy the phospholipid bilayer, thereby undermining the integrity of the BBB (103). At the same time, it stimulates the activation of microglia and releases various inflammatory mediators (104). More importantly, it can mediate apoptosis through two aspects: (1) activation of glutamate-mediated excitatory toxicity (105); and (2) activating the apoptotic cascade to activate the JNK/AP-1 pathway (106).

The anti-inflammatory ILs mainly include IL-2, IL-4, IL-10, IL-13, IL-19 and IL-33 (102). IL-10 has been the focus of neuroprotection after ischemic stroke for a long time. Its protective effect is mainly achieved by inhibiting the inflammatory response. On the one hand, IL-10 reduces the production of proinflammatory mediators by downregulating proinflammatory immune cells, and on the other hand, it upregulates anti-inflammatory immune cells and increases the secretion of neuroprotective factors (107).

Beyond the two classes mentioned above, IL-6 seems to be a special presence. It is also a neurotrophic cytokine, although its serum concentration increases significantly after onset and is associated with poor prognosis (108, 109). IL-6 acts as an inflammatory factor in the acute phase and as a neurotrophic mediator in the subacute and long-term phases.

#### 3.1.2 TNFs

TNF-α may be one of the most widely studied cytokines in the inflammatory response after ischemic stroke. TNF-α can be secreted by a variety of immune cells, but it is mainly secreted by microglia and monocytes after ischemic stroke (110). More specifically, early TNF after permanent MCAO is primarily derived from microglia, while sources are more mixed 12–24 h after permanent MCAO in mice (111–113). TNF-α comes in two forms: a transmembrane form (tTNF-α) that locally regulates inflammation through intercellular interactions, and a bioactive form (sTNF-α) that is soluble and produced by TNF-α invertase.

Numerous studies have shown that TNF-α plays a double-sided role in the pathophysiology of ischemic stroke. While causing nerve damage, it also has a neuroprotective effect (114). TNF-α acts by binding to two different glycosylated receptors (TNFR-1 and TNFR-2). In particular, tTNF-α binds to TNFR-1 and TNFR-2, while soluble TNF-α binds to TNFR-1(101). In animal models, the volume of cerebral infarction is significantly reduced after injection of chimeric monoclonal antibodies against TNF-α (115). Therefore, TNF-α may cause cell damage by binding to TNFR-1. However, injection of cannabidiol into the brain of transient MCAO models show neuroprotective effects by activating the TNF-α/TNFR1/NF-кB pathway (116).

In addition to being involved in neuroinflammatory responses after ischemic stroke, TNF-α entering the bloodstream helps trigger inflammatory cascades. Increased TNF-α concentrations are observed in peripheral blood of stroke patients 6–12 h after symptom onset. Its concentration is directly correlated with NIHSS and infarct size (117, 118). In a long-term follow-up study, serum TNF-α concentrations were associated with poor long-term outcomes after stroke (119). Patient prognosis is associated with TNF-α gene polymorphism (120); therefore, TNF-α inhibition should be considered in the treatment of ischemic stroke.

#### 3.1.3 Chemokines

Chemokines recruit immune cells to sites of inflammation. They are divided into four subfamilies (CC, CXC, XC and CX3C) based on the number and location of cysteine residues. Accordingly, chemokine receptors can be divided into the following categories: CRCCR, CXC-R and CX3C-R (121). It should be noted that the same chemokine can combine with different receptors to play a different role.

After ischemic stroke, the sources of chemokines are diverse, including neurons, astrocytes, microglia, oligodendrocytes and cerebrovascular endothelial cells (122). For example, astrocytes are the primary source of CCL2 in adult stroke models, while damaged neurons are the primary source of CCL2 in neonatal hypoxia/ischemia models (123).

After 24 h of MCAO, CCL2 and CCL3 expression increases in ischemic regions of the MCA (124). CCL2/CCR2 interactions contribute to the recruitment of monocytes and neutrophils after stroke (125). Subsequently, CCL2 expression levels peak at 2 days and decrease after 5 days (126). In the CCR2^−/−^ knockout mouse model, a milder inflammatory response was observed with reduced ischemic infarct loss, reduced cerebral edema, and low BBB permeability (127). CCL3 acts as a proinflammatory chemokine by binding to CCR1 and CCR5 in conjunction with increased expression of CCL2 (128, 129). CCL3 is also an effective chemoattractant for neutrophils (130). It has also been shown to be involved in microglial activation (131). In rat models, the use of antagonists can reduce infarct volume in a dose-dependent manner (132).

In addition to the two chemokines mentioned above, CCL5, as an effective proinflammatory chemokine, selectively binds to three different receptors, CCR1, CCR3 and CCR5 (133). Its proinflammatory effects are associated with leukocyte recruitment and platelet adhesion to the cerebral microvascular system. In a CCL5^−/−^ knockout mouse model of focal cerebral ischemia, there is a significant reduction in infarct volume and improved BBB function (134). Recent studies have shown that CCR5 is important in Treg-cell-dependent BBB protection. CCR5 promotes Treg cells to upregulate PDL1 on the cell surface, which interacts with PD1 expressed on neutrophil surface to inhibit MMP9 expression and protect the BBB (135).

Chemokines and their receptors act as nutritional and protective factors in the nervous system, thereby improving the survival rate of neurons. Importantly, they not only regulate neuronal metabolism but also affect their synaptic transmission (136). Mirabilie-Badenier et al. proposed that CC and CXC chemokines are widely involved in key pathophysiological processes after ischemic stroke (e.g., inflammatory cell recruitment and activation, neuronal survival, and new angiogenesis) and may serve as potential therapeutic targets (137).

### 3.2 Neuroendocrine system

The HPA axis is one of the key components of the stress system (138). Normally, neurons in the paraventricular nucleus secrete corticotropin-releasing hormone (CRH), arginine vasopressin and other neuropeptides that regulate the HPA (139, 140). CRH stimulates the adrenocorticotropic cells in the anterior pituitary to synthesize and secrete adrenocorticotropic hormone (141), which stimulates the adrenal glands to secrete cortisol to regulate normal physiology (142). After stroke, HPA is activated under the stimulation of immune response and pathological stress (143, 144). In particular, inflammatory factors, such as IL-6, released by local inflammation of damaged tissues stimulate the paraventricular nucleus to release CRH (145). In stroke patients, serum IL-6 levels are significantly increased and positively correlated with cortisol levels, suggesting that IL-6 release after cerebral ischemia may contribute to hyperactivation of the HPA (146).

**Figure 2:**
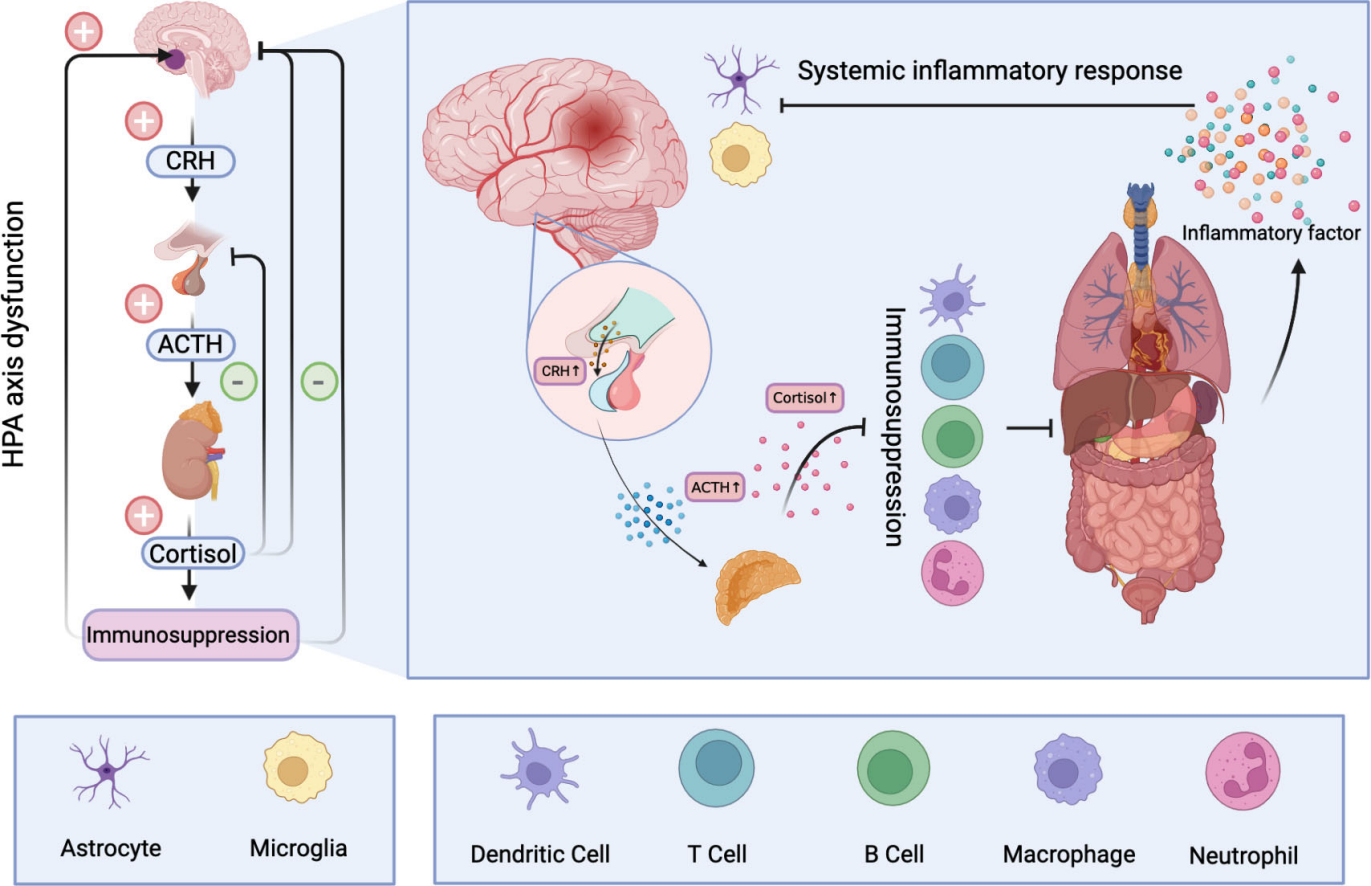
Hyperactivation of the HPA after ischemic stroke results in the release of large amounts of cortisol into the blood. Long-term elevated cortisol inhibits the immune system and can cause dysfunction and even apoptosis of immune cells. A dysfunctional immune system makes peripheral organs vulnerable to damage and produces a large number of inflammatory mediators, resulting in systemic inflammatory response. A storm of inflammatory factors follows. On the one hand, it can further stimulate the HPA, and on the other hand, it disrupts the normal function of nerve cells and has profound effects on the CNS.

Glucocorticoids play an important role in many biological processes such as inflammation and immunity, biological metabolism and water balance (147). Significantly, excess secretion of glucocorticoids has a strong inhibitory effect on the immune system. Glucocorticoids have been shown to inhibit the production of proinflammatory cytokines and proliferation of immune cells, and promote apoptosis (148). In the acute phase, immunosuppression can serve as a protective mechanism that counteracts excessive inflammatory responses to brain damage. However, prolonged immunosuppression increases the risk of infection in patients. Concurrently, glucocorticoid-related toxicity also occurs in multiple organs (149). In the context of systemic inflammatory responses, the HPA is overstimulated and the immune system is further suppressed. Under the vicious circle, the bad clinical outcomes finally appears (150) (**Figure 2**).

### 3.3 Autonomic nervous system

The autonomic nervous system is another key pathway for the CNS to communicate with the periphery. After ischemic stroke, sympathetic and parasympathetic nerves, two important components of the autonomic nervous system, are stimulated and become dysfunctional (151).

#### 3.3.1 Sympathetic hyperactivation

The sympathetic nervous system (SNS) maintains homeostasis during all kinds of physiological activities and prepares the body for emergencies at all times (152). In patients with acute ischemic stroke, autonomic nerve damage, characterized by sympathetic dysfunction, is the most common (153). Plasma catecholamine levels increase significantly in patients with acute brain injury and are inversely correlated with prognosis. The reason behind this may be paroxysmal hypersympathetic syndrome, with clinical features such as hyperthermia, sweating, tachycardia, high blood pressure, shortness of breath, and dystonic posture (154). One of the causes of sympathetic dysfunction in stroke patients is damage to the brain’s norepinephrine-producing nucleus —— locus coeruleus (LC) (155). After the CNS is damaged, the SNS is overactivated and large amounts of norepinephrine are released into the blood (156). Norepinephrine plays a variety of biological functions in the CNS and peripherally through different affinity for α and β adrenergic receptors (ARs) (157–159). In the CNS, the SNS affects attention and memory by regulating neurons, microglia and astrokeratinocytes. Concurrently, it can lead to increased blood pressure and heart rate, and inhibition of gastrointestinal activity (155).

In addition to conventional functions, the SNS regulates the immune system and inflammatory response to protect the body from foreign pathogens and endogenous inflammatory damage factors (156). It is well-known that both αAR and βAR are expressed by both innate and adaptive immune cells, especially β2AR, which is most expressed. After binding to catecholamines, they activate adenylate cyclase through the coupled stimulant protein G (Gαs), leading to an increase in intracellular cAMP levels (160).

In peripheral blood, the continuous elevation of catecholamine can promote apoptosis of lymphocytes and transformation of the Th1 immune response to Th2. These decrease TNF-α levels and the ratio of IFN-γ/IL-4 production (161–164). The SNS releases norepinephrine, which activates β2-AR and limits T cell autoimmunity in the CNS through a mechanism mediated by suppression of IL-2, IFN-γ and granulocyte–macrophage colony-stimulating factor production *via* inducible cAMP early repressor (165). In current clinical practice, β-blockers have been widely used to treat CNS injuries (166). Although β-blockers are effective in reducing mortality, they significantly increase infection rates and require longer ventilator support, intensive care management, and hospital stay (167).

#### 3.3.2 Parasympathetic activation

Previous studies have shown that activation of the parasympathetic nervous system can antagonize various pathological mechanisms. In particular, vagus nerve stimulation (VNS) can effectively improve various brain diseases (168). Continuous VNS increases norepinephrine and acetylcholine (169, 170). Acetylcholine inhibits inflammation through inhibition of the NF-κB pathway mediated by neuronal acetylcholine receptor subunit alpha-7 (nAChRα7) (171). nAChRα7 is an important target for inhibiting the release of proinflammatory cytokines by macrophages and dendritic cells and is expressed in peripheral and CNS macrophages (such as microglia) (172). In response to acetylcholine, macrophages significantly reduce the release of secretory proinflammatory cytokines such as TNF, IL-1β, IL-6 and IL-18, but not anti-inflammatory cytokine IL-10 (171). A recent study showed that vanniclan, a high affinity agonist of nAChRα7, reduces brain inflammation and improved motor function when given to experimental mouse models (173). Although targeting α1/β2-ARs or nAChRα7 may be a novel approach to neuroinflammation, overstimulation of these receptors may increase the risk of infection (156, 174). Animal experiments have shown that electrical stimulation of VN for 15–20 min after transient ischemia significantly reduces extracellular glutamate levels in ischemic tissues (175). VNS significantly inhibits ischemia-induced immune activation and reduces the degree of tissue damage in rats without any reduction in infarct size (176). It is worth noting that VNS can reduce neuroinflammation after ischemic stroke by inhibiting the TLR4/MyD88/NF-κB pathway in microglia while promoting M2 polarization and inhibiting M1 polarization (177).

VNS has been widely used in the postoperative rehabilitation of patients with ischemic stroke. VNS can significantly improve motor function in patients with ischemic stroke, especially in the subchronic stage (178, 179). Therefore, VNS, especially noninvasive VNS, may be a promising adjunctive therapy for ischemic stroke (180).

## 4 The end of the bridge: Systemic immune system disorder

### 4.1 Systemic immunosuppression

During the 24 h following ischemic stroke, circulating levels of cytokines, chemokines and proinflammatory mediators increase in response to systemic immune activation. However, stroke-induced immunosuppression develops 2 days after stroke. The specific manifestations are lymphocytopenia, splenic atrophy and elevated levels of anti-inflammatory cytokines (181, 182). Stroke-induced immunosuppression is considered to be a compensatory mechanism that prevents autoimmunity against CNS antigens (183, 184). Peripheral circulating concentrations of CNS antigens such as myelin basic protein, creatine kinase, neuron-specific enolase, and S100 are significantly elevated within 24 h after stroke. This is consistent with the size of the infarction and associated with higher NIHSS baseline scores (185). Particularly, in response to these CNS antigens, T cells develop a strong cell-mediated inflammatory Th1 type response (186). As the immune system adapts to these antigens, T cells shift from Th1 response to humoral, anti-inflammatory Th2 response to protect the brain from further inflammatory damage and promote tissue repair and neuronal regeneration (187). IL-10 secreted by monocytes, dendritic cells and Treg cells is increased and acts on many immune cell types to avoid proinflammatory responses (188).

Another explanation is that it may be due to abnormal sympathetic nerve function, the parasympathetic nerves and the HPA (163, 189). Sudden increases in circulating levels of norepinephrine and glucocorticoids impair lymphocyte development, transport and function (189). Activation of β-AR inhibits cytotoxic T lymphocyte-associated protein 4 expression on T cells, reduces IFN-γ production and induces apoptosis (163, 190). When propranolol was used to block β-ARs, IFN-γ production was increased and bacterial burden was reduced in mice after stroke (191). The spleen, the largest peripheral immune organ, contracts in response to sympathetic nerve stimulation. Spleen shrinkage may be negatively correlated with stroke infarct volume and there can be marked differences in the size and shrinkage of the spleen among patients of different ages and ethnicities (192, 193). Meanwhile, studies have found that the spleen volume is also negatively correlated with the total number of white blood cells, which may mean an increase in the inflow of white blood cells from the spleen into the blood (194, 195). In tracking splenic cell migration after IS using carboxyfluorescein diacetate succinimide, researchers found that splenic cells such as lymphocytes, monocytes, neutrophils, and natural killer cells could migrate to the brain *via* the circulation (196). However, splenic macrophages/monocytes have been shown to lack very late antigen 4, which prevents these cells from migrating to other tissues. At the same time, the induction of Treg cells and the loss of B cells in the spleen further impair the host-pathogen defense (188).

Previously, it was believed that early splenectomy may alleviate acute brain injury and provide early brain protection. However, some studies have shown that early splenectomy may temporarily improve function but not bring long-term protection to damaged brain tissue. Analogously, delayed splenectomy also brings no benefit to long-term motor and sensory function recovery (197).

### 4.2 Opportunistic infection

**Figure 3:**
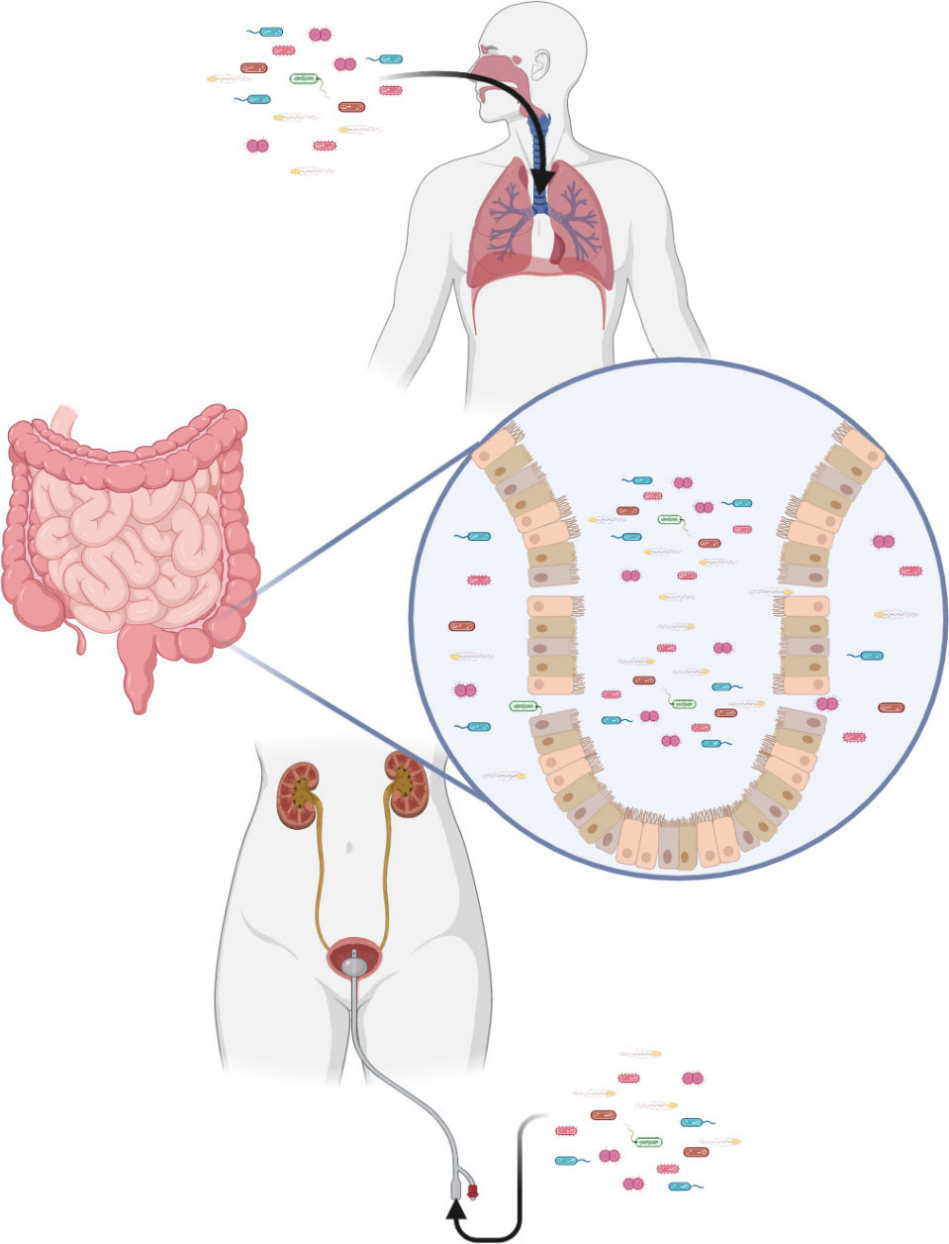
Immunosuppression after stroke increases the risk of infection. Exogenous pathogens can infect the lungs and urinary tract. Translocation of intestinal flora is also a potential risk.

Severe peripheral immunosuppression makes the body vulnerable to both exogenous and endogenous pathogens (**Figure 3**). Pneumonia and urinary tract infection are the main types of exogenous infection after stroke. The former has a high incidence of 57%, while the latter has an incidence of 11–27% (198). In particular, stroke-associated pneumonia (SAP), the most common type of infection after stroke, often indicates deterioration of the disease and poor prognosis (199). There are currently two definitions of SAP. One is described as pneumonia occurring < 3 days after stroke. A broader definition classifies SAP as acute (when pneumonia occurs within 1 month of a stroke) and chronic (when it occurs after 1 month). Clinically, SAP is usually defined using broad criteria (200). Through numerous clinical studies, aspiration and its associated risk factors, such as impaired levels of consciousness and dysphagia, have been identified as important risk factors for SAP (200, 201). The immunosuppression that accompanies ischemic stroke also contributes to lung injury (202).

The intestinal flora that co-exists with us and the viruses that lurk in the body are predictable consequences of immunosuppression after stroke (203). Compared with healthy people, the gut microbiota is significantly dysregulated in stroke patients, manifested as having more bacteria producing short-chain fatty acids, such as *Odoribacter*, *Akkermansia*, Ruminococcaceae_UCG_005 and *Victivallis* (204). Short-chain fatty acids produced by these bacteria induce differentiation of naive T cells towards the functional Treg cell phenotype and away from the proinflammatory Th17 phenotype (205). Although Treg cells are closely related to neuroprotective function by inhibiting pathogenic T cells, some cytokines produced and secreted by they may associated with neurotoxicity (206, 207). In addition, species diversity of intestinal microecology decreases after stroke (208). Specifically, alterations are observed within the highly abundant phyla Firmicutes, Bacteroidetes and Actinobacteria (209). Immunosuppression creates conditions for the displacement of intestinal flora and causes infection (203).

## 5 Conclusion

Ischemic stroke is not only an acute and severe neurological disease but also a multiorgan and systemic disease. Throughout the course of the disease, immunoreactivity is involved at every stage.

Initially, the innate immune system in the CNS is activated in response to ischemia–reperfusion injury of brain tissue. As the disease progresses, the large number of inflammatory mediators produced by neuroinflammatory responses activate the adaptive immune system to further damage nerve cells. In this process, in addition to the physical function of the damaged brain area, the inflammatory factors produced by neuroinflammation interfere with other normal neural pathways. In particular, dysfunction of the HPA and autonomic nervous system affects the function of the immune system and peripheral organs. They not only cause immune disorders but also lead to dysfunction of peripheral organs. Eventually, all of these feed back into the CNS and lead to a vicious cycle.

In theory, regulating the immune system or blocking inflammatory pathways from the center to the periphery is a promising therapy for improving the prognosis of ischemic stroke. However, with current technology, it is difficult to reduce systemic inflammation without increasing the risk of endogenous and exogenous infection. In addition, the neuroendocrine axis and autonomic nervous system are important pathways linking the center and the periphery. They also play an important role in regulating immune system function. Although researchers have been working in this area for a long time, the detailed mechanism remains unclear. These may be breakthrough points for designing treatment strategies in the future.

## Author contributions

FW and ZL conceived the framework and wrote the manuscript. FW, LZ, and DY were responsible for the illustrations. YZ, KH, YW, and XX were responsible for the compilation and editing of the literature. JS and RZ were responsible for the review and revision of the article. All authors contributed to the article and approved the submitted version.

## Funding

The research was funded by the National Natural Science Foundation of China (82071285) and the Zhejiang Medical and Health Science and Technology Plan (G3222234).

## Acknowledgments

Figures were created with the help offered by BioRender.com.

## Conflict of interest

The authors declare that the research was conducted in the absence of any commercial or financial relationships that could be construed as a potential conflict of interest.

## Publisher’s note

All claims expressed in this article are solely those of the authors and do not necessarily represent those of their affiliated organizations, or those of the publisher, the editors and the reviewers. Any product that may be evaluated in this article, or claim that may be made by its manufacturer, is not guaranteed or endorsed by the publisher.
